# Dynamic Ultrasound Projector Controlled by Light

**DOI:** 10.1002/advs.202104401

**Published:** 2022-01-24

**Authors:** Zhichao Ma, Hyungmok Joh, Donglei Emma Fan, Peer Fischer

**Affiliations:** ^1^ Max Planck Institute for Intelligent Systems Heisenbergstr. 3 Stuttgart 70569 Germany; ^2^ Materials Science and Engineering Program Texas Materials Institute The University of Texas at Austin Austin TX 78712 USA; ^3^ Walker Department of Mechanical Engineering The University of Texas at Austin Austin TX 78712 USA; ^4^ Institute of Physical Chemistry University of Stuttgart Pfaffenwaldring 55 Stuttgart 70569 Germany

**Keywords:** acoustic hologram, acoustic wavefront control, light‐addressable electrochemistry, microbubble generation, ultrasound modulation

## Abstract

Dynamic acoustic wavefront control is essential for many acoustic applications, including biomedical imaging and particle manipulation. Conventional methods are either static or in the case of phased transducer arrays are limited to a few elements and hence limited control. Here, a dynamic acoustic wavefront control method based on light patterns that locally trigger the generation of microbubbles is introduced. As a small gas bubble can effectively stop ultrasound transmission in a liquid, the optical images are used to drive a short electrolysis and form microbubble patterns. The generation of microbubbles is controlled by structured light projection at a low intensity of 65 mW cm^–2^ and only requires about 100 ms. The bubble pattern is thus able to modify the wavefront of acoustic waves from a single transducer. The method is employed to realize an acoustic projector that can generate various acoustic images and patterns, including multiple foci and acoustic phase gradients. Hydrophone scans show that the acoustic field after the modulation by the microbubble pattern forms according to the prediction. It is believed that combining a versatile optical projector to realize an ultrasound projector is a general scheme, which can benefit a multitude of applications based on dynamic acoustic fields.

## Graphical abstract

Dynamic wavefront control of ultrasonic waves is fundamental for many acoustic technologies. Conventional methods are either static or possess a limited number of active elements resulting in coarse images. A high‐resolution light‐controlled dynamic ultrasound projector is demonstrated. Low‐intensity light switches a photoconductor and locally triggers electrochemical reactions to form microbubble patterns. These enable dynamic ultrasound focusing and phase gradients.

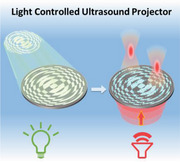

## 1 Introduction

Wavefront control underlies several important developments in telecommunications,^[^ ^1^, ^2^ ^]^ manufacturing,^[^ ^3^, ^4^ ^]^ and imaging.^[^ ^5^, ^6^ ^]^ For example, devices used to shape an optical wavefront, such as a spatial light modulator (SLM) or a digital mirror device (DMD), are widespread as they can be found in many optical projection systems. They have also enabled optical applications in science.^[^ ^7^, ^8^ ^]^ SLMs and DMDs have benefitted from the tremendous progress in display technology and micro‐electromechanical systems. SLMs control the phase and amplitude of an optical wave by electrically switching the birefringence in each pixel of a liquid crystal display. The DMD is a micro‐electromechanical device where individual micromirrors are switched to control the intensity at each mirror (pixel). Both can be integrated into chip‐sized devices. SLMs can be used to tune the phase and amplitude of an entire optical image (with several megapixels). Similarly, DMDs can be used to project amplitude images and mix different color channels. In comparison, acoustic waves have no polarization and show little dispersion across the wide frequency range from audible to ultrasound frequency, thus making it difficult to dynamically control acoustic wavefronts in an approach similar to an SLM. DMDs operate in air and can thus also not readily be adapted for the modulation of underwater acoustic fields.

Wavefront control in acoustics has thus largely relied on traditional methods such as an acoustic lens or phased transducer arrays for beam focusing or the generation of phase gradients, which have been exploited in sensing,^[^ ^9^ ^]^ imaging,^[^ ^10^ ^]^ and manipulation.^[^ ^11^, ^12^ ^]^ However, there are clear limitations to these conventional approaches for acoustic wavefront shaping. For example, the acoustic lenses are static.^[^ ^13^, ^14^ ^]^ The phased transducer array is in principle capable of generating acoustic waves with tunable wavefronts, but the complexity of its electrical driving circuitry is high and the mechanical coupling between the transducer elements limits the number and density of transducers and hence the complexity of the acoustic images that can be generated. Recently, Melde et al.^[^ ^15^ ^]^ have demonstrated how the phase of MHz acoustic fields can be shaped with 3D‐printed acoustic phase plates (holograms). In combination with a linear phased transducer array,^[^ ^16^ ^]^ it is possible to impart some dynamic variation to the phase plate. Besides, acoustic metamaterials have been considered to address the challenge of forming reconfigurable wavefronts.^[^ ^17^, ^18^ ^]^ However, especially for underwater ultrasound (MHz frequencies), it is challenging to manufacture the necessary subwavelength structures. For applications in liquids at MHz frequencies, we have recently developed a spatial ultrasound modulator that is based on digitally controlled (electronically triggered) microbubble arrays. They can be used to dynamically reshape the incident plane ultrasonic waves into complex amplitude distributions.^[^ ^19^ ^]^ Briefly, a complementary metal‐oxide‐semiconductor chip, which has 10 000 individually addressable electrodes, is used for the electrolysis of water to generate microbubbles, which locally block the transmission of ultrasound. Even though it only takes microseconds for bubbles to form, the total processing time was approximately seconds. To realize faster dynamic wavefront control over larger areas and with more elements, it is necessary to develop a highly parallel, high‐resolution scheme to modulate ultrasound wavefronts.

Here, we demonstrate how complex optical images or light patterns (with millions of pixels), that are readily generated with SLMs or DMDs, can be used to realize the fast and parallel control of ultrasound fields. We use optical images projected onto photoconductive substrates to locally generate microbubbles in >10 000 locations in parallel within ≈100 ms. We show that this large number of tunable effective ultrasound elements can be used for dynamic beam focusing and the generation of acoustic wavefronts with a phase gradient.

## 2 Results and Discussions

### 2.1 Photo‐Addressable Microbubble Generation

**Figure 1:**
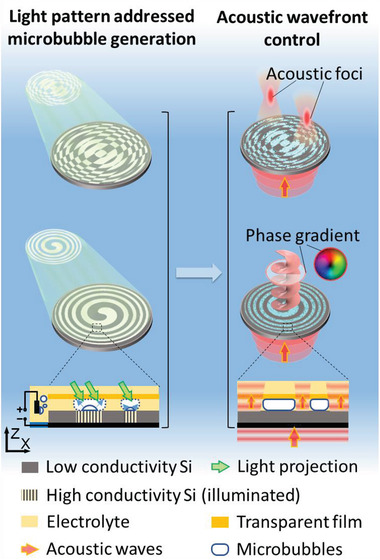
Schematic of the light pattern addressable microbubble generation and its applications in acoustic wavefront control. Left: the light pattern is projected on the silicon wafer to locally increase its conductivity, and thus generates microbubble patterns based on electrochemical reactions. Right: The microbubble pattern modulates the incident plane acoustic waves into acoustic foci or generates an acoustic phase gradient, as the microbubbles have a significant acoustic impedance mismatch with the surrounding liquids.

The acoustic impedance mismatch between gas and liquid is so large that a thin layer of gas can effectively block the transmission of ultrasound.^[^ ^20^ ^]^ Thus, a microbubble pattern in the path of an ultrasound wave could be used to block ultrasound everywhere there are bubbles and thus control the wavefront by amplitude modulation. Microbubbles can be generated by direct thermal heating^[^ ^21^ ^]^ or via a photo‐thermal mechanism,^[^ ^22^ ^]^ then one would require high intensities, which would be impractical. Thus, electrochemical reactions are more efficient.^[^ ^23^ ^]^ Here, we use a light pattern to define the locations on an electrode where bubble‐forming electrochemical reactions take place (**Figure** **1**). The large‐area (≈7 cm^2^) and fast (≈100 ms) electrochemical microbubble generation over the entire surface can be controlled with a light pattern. In contrast, conventional direct photo‐thermal bubble generation would require orders of magnitude higher light intensities. Our approach therefore presents a major simplification, as light patterns can be easily generated. Furthermore, it avoids complex electrical circuitry and the light is only used to trigger the electro‐chemical bubble generation. Our scheme is thus suitable for large areas and makes it possible to project ultrasound beams.

Our ultrasound projector is shown in Figure 1. The light controllable microbubble generation is based on a photoconductive substrate, whose conductivity is reversibly tuned upon illumination with light. The top side of the wafer is immersed in an electrolyte, in which the electrical current will induce water electrolysis to produce gas bubbles. A voltage (31 V) is applied between the bottom side of the wafer and the opposite electrode, which sits in the electrolyte. In the case of no illumination, the electrical conductivity across the wafer is low (≈50 mS cm^–1^), and thus the applied voltage (and current) cannot drive the electrochemical reaction. However, as soon as the light is directed onto the wafer, the wafer becomes conductive at the site of illumination, so that the current through the wafer increases and locally generates microbubbles at the wafer–electrolyte interface. The optical image thus directly defines the microbubble pattern, which in turn is used to shape and modulate the incident acoustic wave. There is a transparent polyester film covering the silicon wafer to keep the microbubbles in place. As shown in our previous work,^[^ ^19^ ^]^ the microbubbles are held in place even when exposed to high‐intensity ultrasound of 5 W cm^–2^. The microbubble pattern can be removed by flow jetting or mechanical wiping, such that a new acoustic image can be projected. Compared to the phase modulation method,^[^ ^15^ ^]^ the amplitude hologram blocks parts of the beam which reduces the available power, but in our implementation enables dynamic control.

**Figure 2:**
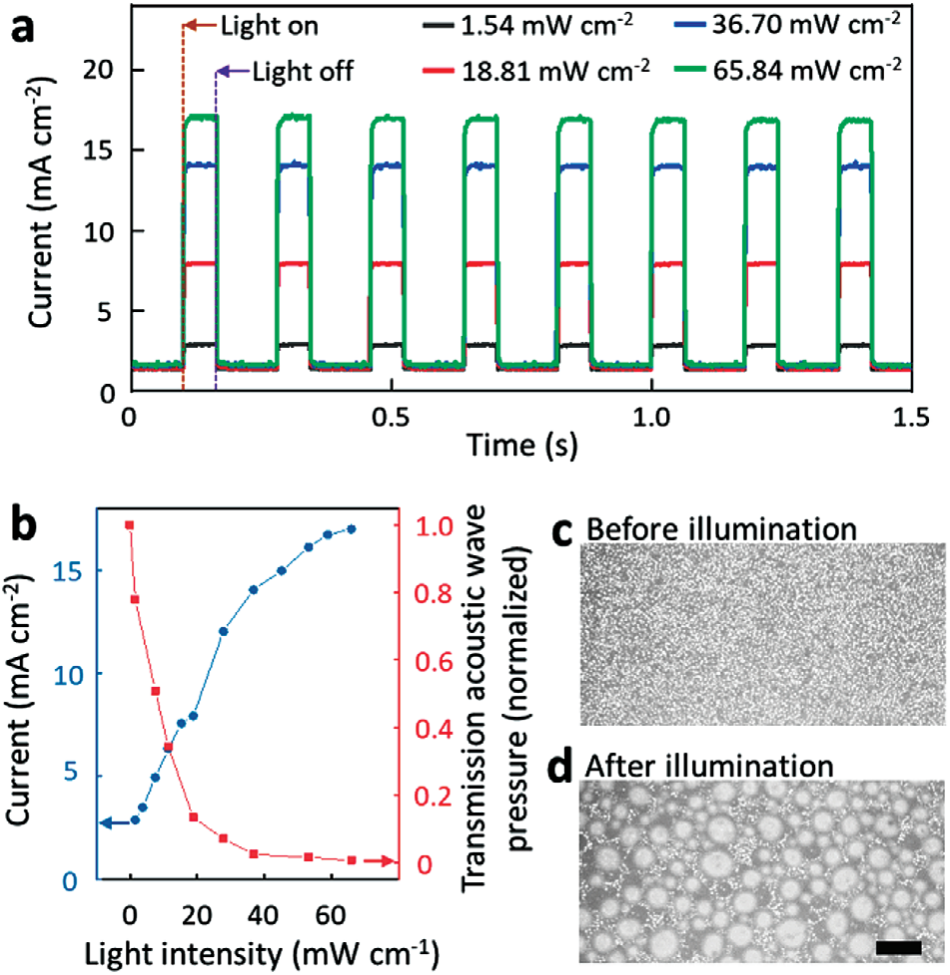
a) The electrical current through the silicon wafer under a certain voltage indicates the reversible alternative change between low conductivity state (no illumination) and high conductivity state (illumination). b) As the light intensity projected on the wafer surface increases, the photocurrent increases, and the transmission acoustic pressure decreases due to the boost of the microbubble generation. Microscopic images of c) the silicon wafer surface before illumination and d) the generated microbubbles on the silicon wafer surface after illumination. The light intensity was 65 mW cm^–2^ and the projection time was 0.1 s. The scale bar is 0.2 mm.

We used p‐type‐doped silicon wafers as the photoconductive layer for the light‐addressed microbubble generation. The photoconductivity of p‐type‐doped silicon has found application in light‐addressed neural stimulation^[^ ^24^ ^]^ as well microparticle manipulation.^[^ ^25^ ^]^ In our setup, the bottom surface of the doped silicon wafer is grounded with a silver epoxy coating, while the top side is immersed in the electrolyte, where the positive electrical potential resides. The light patterns used to switch the p‐doped Si wafer are generated with a digital mirror device (DMD). Light illuminates the DMD and the desired pattern is projected onto the silicon wafer. **Figure** **2** shows the photoconductivity of the wafer as a function of the light intensity and duration of illumination. As shown in Figure 2a, the electrical current through the wafer strongly increases upon illumination, and reversibly switches back to a negligible dark current when the illumination is off. The increase in the electrical current (Figure 2b, blue curve) causes a corresponding increase in the electrolysis and hence the generation of hydrogen bubbles at the wafer surface. The rate with which gas is generated at the wafer surface is estimated as 93 nmol cm^–2^ s^–1^ for an incident light intensity of ≈66 mW cm^–2^.

To estimate the ability of the bubbles to block ultrasound, we monitored the acoustic pressure with a hydrophone that is placed 50 mm atop the wafer surface. The transmission of a 5 MHz wave is seen to decrease as the projected light intensity increases (Figure 2b, red curve). At a light intensity of around 30 mW cm^–2^, the acoustic transmission is tenfold smaller in comparison to no light projection, indicating the effective blocking of the acoustic waves by the microbubbles. Figure 2c,d, respectively, shows photographs of the wafer surface before and after the generation of microbubbles (the illumination is 65 mW cm^–2^ projection for 0.1 s). The microbubble generating process is also shown in Video S1 in the Supporting Information.

### 2.2 Acoustic Focusing via Light‐Controlled Microbubble Patterns

As a first example, we consider the generation of controlled acoustic foci. Focusing of ultrasound is important for biomedical imaging, nondestructive testing, hyperthermia, and neural modulation. These applications all benefit from dynamically tunable acoustic foci. Conventional acoustic lenses are generally static, and phased transducer arrays do not have many elements that can give rise to sophisticated pressure distribution. Both shortcomings can be overcome with the approach we describe herein.

**Figure 3:**
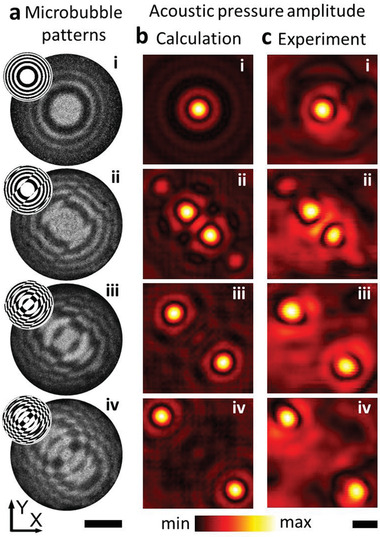
Acoustic focusing based on the light addressed microbubble generation. a) The microbubble pattern generated on the silicon surface. The images were obtained by subtracting the photo with that before illumination. The scale bar is 10 mm. The projected light patterns are in the upper left insets. The microbubble generation process is shown in Video S2 in the Supporting Information. b) The calculated acoustic field at the target focus plane (80 mm away from the silicon wafer surface). c) The experimental results reveal the microbubble pattern modulates the acoustic waves into target foci. The scale bar is 2 mm in (b) and (c).

We show that changing the light pattern also shifts the acoustic foci to new locations, as shown in **Figure** **3**. For this, we first compute the required binary amplitude acoustic pressure patterns (see the Experimental Section for details concerning the calculations). The calculated target bubble patterns (shown in the insets of Figure 3a) are then used as input images for the digital mirror device. The light pattern projection via the DMD is turned on for 100 ms, during which the p‐type‐doped silicon locally becomes conductive. The corresponding microbubble patterns correspond to the optical image, as can be seen in Figure 3a. A 5 MHz acoustic wave is passed through the silicon wafer from the transducer situated beneath the silicon wafer. The acoustic wave is locally blocked where there are microbubbles and this modulates the wavefront. The projected acoustic fields have been mapped with a hydrophone scan, as shown in Figure 3, and the resulting acoustic pressure distributions are in excellent agreement with the numerical calculation (Figure 3b,c).

### 2.3 Acoustic Phase Gradient Generation

**Figure 4:**
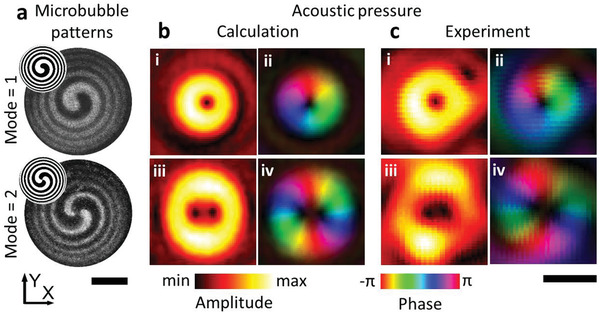
Acoustic phase gradient generation based on the microbubble pattern. a) The microbubble pattern generated on the silicon surface. The images were obtained by subtracting the photo with that before illumination. The scale bar is 10 mm. The projected light patterns are in the upper left insets. The microbubble generation process is shown in Video S2 in the Supporting Information. b) The calculated acoustic fields and the acoustic phase gradient at ii) 1st mode and iv) 2nd mode. c) The experimental results reveal the microbubble pattern modulates the acoustic waves to the target phase gradient. The areas of high pressure in the amplitude have been highlighted in the acoustic phase field (b‐ii, iv) and (c‐ii, iv). The scale bar is 2 mm in (b) and (c).

Beyond amplitude field modulation, it is also essential to be able to affect the phase of a wave. For example, an acoustic vortex beam is formed by a phase gradient and it is especially useful for particle manipulation^[^ ^26^, ^27^, ^28^ ^]^ and underwater communication.^[^ ^29^, ^30^ ^]^ Conventional methods used to generate an acoustic vortex beam include static phase masks,^[^ ^14^, ^31^ ^]^ structured transducers,^[^ ^28^, ^32^ ^]^ and phased transducer arrays.^[^ ^12^, ^30^ ^]^ However, it remains challenging to realize dynamic and quickly reconfigurable vortex fields. Here, we show that light‐images can generate microbubble patterns which in turn cause the incident plane acoustic wave to form an acoustic vortex beam, as shown in **Figure** **4**. For each target phase gradient (modulus = 1 or 2), the microbubble patterns were first computed as shown in the insets of Figure 4a. The microbubble patterns are then generated by the corresponding light patterns (Figure 4a). The numerical simulations predict the acoustic vortex fields depicted in Figure 4b, and the hydrophone scan shows the experimental results (Figure 4c). Both the amplitude distribution and the phase gradient agree well with the numerical simulation, demonstrating the utility of our ultrasound projection system. As shown in our previous study,^[^ ^19^ ^]^ the microbubble patterns can be mechanically wiped off within a second. Clearing the bubbles much faster, for instance with flow, will enable fast transitions between different acoustic modes.

## 3 Conclusion

In summary, we have demonstrated the light‐addressable generation of microbubble patterns for the dynamic control of acoustic wavefronts. The microbubble pattern can modulate an incident plane acoustic wave that diffracts into target acoustic foci or controlled beams shaped by the induced phase gradients. Our scheme is fast, versatile, with very high resolution, and easily scalable to large sizes, as the light‐triggered microbubbles are generated within 100 ms, and as their distribution is simply controlled by the projection of a low‐intensity optical image (65 mW cm^–2^, active elements >10 000). No high‐power lasers or complex electrical circuitry is needed. We experimentally validated the acoustic fields and show that the experimentally obtained pressure distributions are in excellent agreement with simulation results. We expect that our device could be used in the frequency range between 100 kHz and 10 MHz, where the lower limit is given by a sufficiently low acoustic transmission (high blocking) due to the microbubble layer and the upper limit due to the size of the microbubbles. Currently, the bubble patterns are used to modulate the acoustic wave itself. Future research is expected to look into nonlinear acoustic effects, such as cavitation in transient high‐intensity ultrasound, where beam modulation could aid biomedical applications. The modulated ultrasound waves could also be used for biological samples, e.g., cell patterning^[^ ^33^ ^]^ and tweezing.^[^ ^28^ ^]^ Toward these applications, long‐term acoustic excitation from minutes to hours might be required. While we did not observe any changes to the microbubble patterns during the hydrophone scan that lasted for 60 min, future studies should explore further stabilization of bubbles, possibly utilizing suitable interface treatments. To meet the requirement of a durable acoustic modulation, the method of mechanically wiping the microbubbles from the wafer should be replaced with either hydrodynamic flow or by running the back reaction in electrolysis,^[^ ^34^ ^]^ to prevent abrasion of the silicon surface. The light addressable microbubble generation extends the capabilities of acoustic wavefront control and will be essential for sophisticated ultrasound applications ranging from microparticle manipulation to biomedical imaging and medical ultrasound.

## 4 Experimental Section

### Sample Preparation and Acoustic Setup

The photoconductive wafers used for the light pattern addressed bubble generation were p‐type (Boron) doped silicon wafers (Silicon Materials, Germany). The wafer had a diameter of 50.8 mm and a thickness of 275 µm. Its electrical resistivity in a dark field was ≈20 Ω cm. One side of the wafer was polished. On this side, a silver‐filled epoxy paste layer (Epoxy Technology Inc., MA) was manually applied. The wafer was baked at 65 ℃ for 1 h to harden the coated silver epoxy. The wafer was then positioned at the bottom of a water tank, whose bottom surface had an opening for the silver epoxy coated area. The tank was then filled with electrolyte (80 mg mL^−1^ aqueous K_2_SO_4_ solution). The top side of the silicon wafer (roughness ≈0.7 µm) faced the electrolyte solution. A DC power supply provided 31 V between the silver epoxy layer (cathode) and the anode (immersed steel post). A transparent polyester film with a thickness of 90 µm covered the silicon wafer to keep the microbubbles in place. The microbubble patterns could be mechanically wiped off to permit the generation of a new pattern, as shown in a previous study.^[^ ^19^ ^]^

A 5 MHz transducer (I8‐0518‐P, Olympus Corporation, Japan) was placed at the bottom of the silicon wafer. Between the transducer and the silicon wafer, a thin glycerol layer was used as an ultrasound coupling agent. The transducer was driven with a 10 Vpp electrical signal. A needle‐type hydrophone (0.5 mm diameter, Precision Acoustics Ltd., UK) was used to measure the acoustic field at a distance of 80 mm away from the silicon wafer. The signal from the hydrophone was amplified and filtered by a lock‐in amplifier (Zurich Instruments, Switzerland). The scan area was 10 mm × 10 mm with a scan step size of 0.1 mm. It should be noted that the hydrophone tip was relatively large and that a subwavelength hydrophone tip would reveal greater detail in the acoustic pressure fields.

### Optical Setup

The dynamic light pattern projected onto the silicon wafer was generated with a spatial light projection system (Figure S1, Supporting Information). The light source was a 532 nm laser beam (Verdi G10, Coherent), which was expanded and directed on a digital mirror device (DMD; V‐7000, ViaLUX GmbH, Germany). The DMD had an array of 1024 × 768 mirrors (pixels), which selectively reflected the light beam into patterns according to a software input. The reflected light pattern was then directed to the silicon wafer with a 45° angle of incidence to locally control the wafer's electrical conductivity. The projection area on the wafer surface was 29 × 29 mm^2^, corresponding to 432 × 306 elements at the DMD. Every pixel (micromirror) of the optical modulator illuminated an area of 67 µm × 95 µm on the Si wafer. Within this area, many microbubbles were generated with differing sizes, so that the optical pixel size did not correspond to the microbubble size. The acoustic pressure pattern shown in Figures 3 and 4 was formed with ≈16 000 pixels per cm^2^ of the acoustic image. To determine the light intensity projected onto the wafer, a thermal power sensor (S310C, Thorlabs Inc., NJ) was placed at the silicon wafer projection site with all the pixels of the DMD set to project light to the wafer.

### Acoustic Field Calculation

The design of the microbubble patterns was based on the calculation of a propagating acoustic wave. The microbubble generation surface was set as the *X*–*Y* plane, which was at the *Z*‐coordinate at the origin. At the center of this plane, an array of 432 (*X*) × 306 (*Y*) elements was defined as the projection region, which corresponded to an area of 29 × 29 mm^2^. In this study, the target focus points were all set at the *X*–*Y* plane at *Z* = 80 mm. The phase delay for each element in the projection area was mapped based on their distance from the target focus point. In the case of multiple foci, the phase delays from different foci were averaged. In the case of the vortex beam generation, a spatial angular momentum was chosen as the phase delay for each element. Based on the calculated phase map, the areas with a phase within −*π*/2 to *π*/2 were defined as bubble‐free, which were dark in the light pattern; while the other areas were set to be covered with bubbles and were bright in the light pattern. These patterns were then transferred to the DMD to project the corresponding image. To simulate the acoustic propagation through the microbubble pattern, the angular spectrum method was used to calculate the acoustic propagation from the silicon wafer surface. The areas covered by microbubbles were set to have zero‐acoustic pressure, while the uncovered areas were all set to have the same acoustic pressure and phase as the incident plane wave from the transducer.

## Conflict of Interest

The authors declare no conflict of interest.

## Supporting information

Supporting Information

Supplemental Video 1

Supplemental Video 2

## Data Availability

The data that support the findings of this study are available from the corresponding author upon reasonable request.
