# Supplementary material for: Dynamic Ultrasound Projector Controlled by Light
Source: Adv Sci (Weinh). 2022 Jan 24;9(9):2104401. doi: 10.1002/advs.202104401 (PMC8948597; doi:10.1002/advs.202104401)
Supplement: Supplementary file 1 — Supporting Information [file ADVS-9-2104401-s001.pdf]

WILEY-VCH

ADVANCED SCIENCE
Open Access

Supporting Information

for *Adv. Sci.,* DOI: 10.1002/advs.202104401

Dynamic ultrasound projector controlled by light

Zhichao Ma, Hyungmok Joh, Donglei Fan, Peer Fischer**

Supporting Information

Dynamic ultrasound projector controlled by light

Zhichao Ma, Hyungmok Joh, Donglei Fan, Peer Fischer**

Supplementary Figure

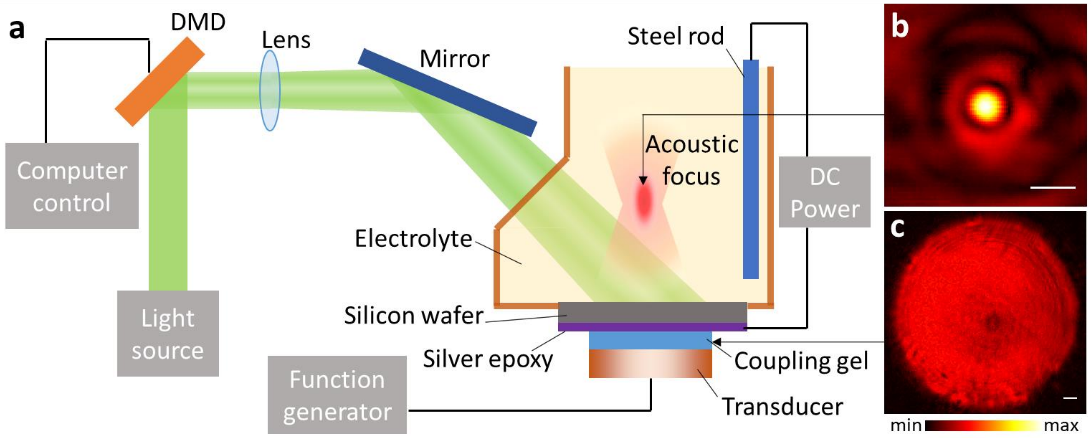

Figure S1. (a) The schematic of the experimental setup. The acoustic pressure scan shows an acoustic focus at the target plane (b), which is modulated from the plane acoustic waves emitted by the transducer (c). The hydrophone scan of (c) is conducted in a water tank, in which the transducer is immersed. The scale bars are both 2 mm in (b) and (c).

Supplementary Table

Table S1. The acoustic properties of the components used in the dynamic ultrasound projection system.

Component	Material	Density (kg m^{-3})	Speed of Sound (m s^{-1})	Acoustic Impedance (MRayl)
Coupling gel	Glycerol	1260	1964	2.5
Photoconductive layer	Silicon	2330	8433	19.6
Electrolyte	K_2SO_4 solution	1060	1500	1.6
Microbubble	Hydrogen	0.089	1300	1.16×10^{-4}

Experimental videos

Video S1. Microbubble generation via light controlled electrochemical reactions. From 0 s to 10 s of the video, the DC power was turned on but without laser light projection. The bright white spots are the reflections of the background illumination by the unpolished silicon wafer. At 10 s, the laser light was projected onto the silicon wafer surface for 0.1 s. The bubbles were then generated by the light controlled electrochemical reaction.

Video S2. Light pattern addressed bubble generation. At 1.0 s of the video, the light patterns modulated by the optical system were projected on the wafer for 0.1 s. Then the corresponding bubble pattern was generated.
